# Molecular basis of nucleotide-dependent substrate engagement and remodeling by an AAA+ activator

**DOI:** 10.1093/nar/gku588

**Published:** 2014-07-25

**Authors:** Vidya C. Darbari, Ed Lawton, Duo Lu, Patricia C. Burrows, Simone Wiesler, Nicolas Joly, Nan Zhang, Xiaodong Zhang, Martin Buck

**Affiliations:** Department of Life Sciences, Imperial College London, London SW7 2AZ, UK

## Abstract

Binding and hydrolysis of ATP is universally required by AAA+ proteins to underpin their mechano-chemical work. Here we explore the roles of the ATPase site in an AAA+ transcriptional activator protein, the phage shock protein F (PspF), by specifically altering the Walker B motif sequence required in catalyzing ATP hydrolysis. One such mutant, the E108Q variant, is defective in ATP hydrolysis but fully remodels target transcription complexes, the RNAP-σ^54^ holoenzyme, in an ATP dependent manner. Structural analysis of the E108Q variant reveals that unlike wild-type protein, which has distinct conformations for E108 residue in the ATP and ADP bound forms, E108Q adapts the same conformation irrespective of nucleotide bound. Our data show that the remodeling activities of E108Q are strongly favored on pre-melted DNA and engagement with RNAP-σ^54^ using ATP binding can be sufficient to convert the inactive holoenzyme to an active form, while hydrolysis *per se* is required for nucleic acid remodeling that leads to transcription bubble formation. Furthermore, using linked dimer constructs, we show that RNAP-σ^54^ engagement by adjacent subunits within a hexamer are required for this protein remodeling activity while DNA remodeling activity can tolerate defective ATP hydrolysis of alternating subunits.

## INTRODUCTION

In AAA+ proteins a cycle of nucleotide (ATP) binding and hydrolysis allows the formation of at least two different conformations, each associated with a distinctive functional state (1). AAA+ proteins often exist as hexamers and inter-subunit coupling allows the propagation of conformation from one active site to others within an AAA+ assembly (2). The ATP-dependent conformational coupling can support various activities of AAA+ assemblies, including that of a bimodal switch and a processive machine (3,4). The former is often associated with slow ATP hydrolysis, the latter with multiple rounds of coupled binding and hydrolysis events. AAA+ proteins share conserved functional motifs including those of Walker A for nucleotide binding, Walker B and Arginine fingers for ATP hydrolysis.

Bacterial enhancer binding proteins (bEBPs) are specialized AAA+ proteins that are required to activate the sigma54-dependent transcriptional complexes (5,6). Sigma54 (σ^54^) recruits RNA polymerase (RNAP) to specific promoter sites and forms a stable closed complex (RP_c_) unable to proceed to intermediate states (RP_i_) or the open complex (RP_o_) used in transcription initiation. As transcriptional activators, bEBPs function to catalyze the conversion of RP_c_ to RP_o_, causing the RNAP-σ^54^ holoenzyme to isomerize and promoter DNA to melt, leading to transcription initiation (7,8,9,10). Formation of RP_o_ is associated with large-scale conformational changes in RNAP-σ^54^, and these are inhibited by sigma54 prior to the remodeling by bEBP activators (11–15).

bEBPs can exist in a number of distinct functional and conformational states including that of nucleotide free (APO), ATP bound, transition state (as mimicked by ATP transition state analogue ADP.AlF), and ADP bound (16–19). Similar to other ATPases, ATP binding and hydrolysis involve distinct kinetic steps including ATP binding, gamma-beta bond cleavage, Pi release and ADP release. Hexameric bEBPs are shown to stably bind sigma54 as well as RP_c_, only when non-hydrolysable ATP or ATP hydrolysis transition state analogues are present or when the ATP is very slowly hydrolyzed (17,20–22). However, under such conditions either none or an incomplete remodeling of RP_c_ is observed, and the *de novo* DNA melting characteristic of RP_o_ formation is not observed (23–25). Full remodeling of RP_c_ and formation of RP_o_ was therefore suggested to depend on ADP + Pi formation and/or Pi release (23,26).

**Figure 1.**
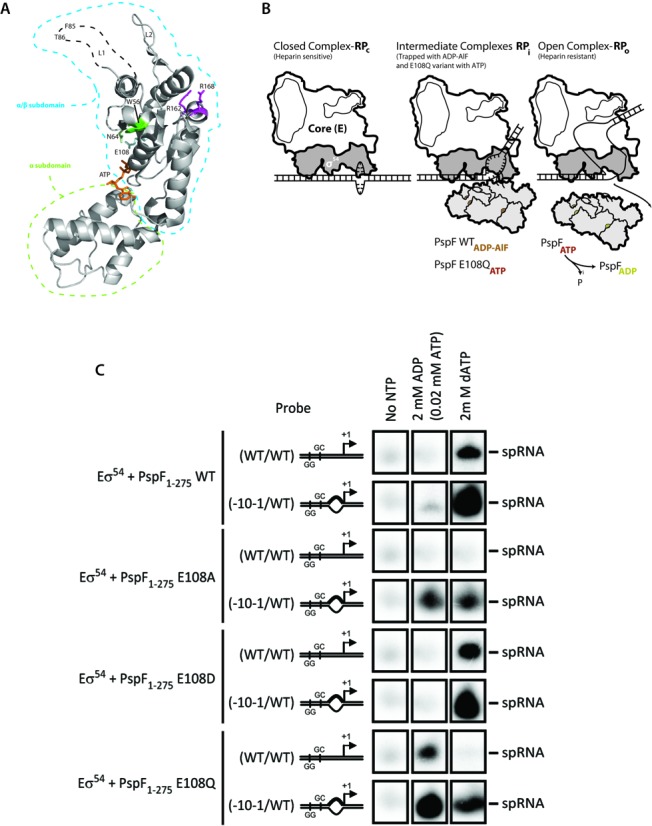
(**A**) Crystal structure of PspF AAA domain with key residues labeled. (**B**) Cartoon depicting the transition from RP_c_ to RP_o_ with intermediates RP_i's_ (either WT PspF trapped with ADP-AlF or PspF E108Q variant in complex with ATP). (**C**) Transcriptional activities of E108 variants (E108A, E108D and E108Q) and wild-type protein. Different nucleotides are assayed with duplex DNA or DNA with pre-opened transcription bubbles.

A number of important structural and functional motifs within bEBPs have been identified and characterized, including the signature GAFTGA motif, which is almost invariant among bEBPs and directly engages with the RP_c_ substrate, and the ‘Glutamate Switch’, a common signature in AAA+ proteins (Figure 1A). The ‘Glutamate Switch’ pair (E-N) was identified originally in PspF as the E108 side chain adopts different conformations in the presence of ATP or ADP, forming or interrupting the interactions with N64 (3,11,16,22,27). These differences are correlated with the distinct conformations of the GAFTGA containing L1 loop inserted into the AAA+ domain, determining whether or not L1 could bind its target substrate σ^54^ in RP_c_ (Figure 1A). In the ADP form, E108 does not interact with N64 and the L1 loop is in a ‘locked’ conformation that is unable to interact with its substrate. While in the ATP or ADP.AlF transition state, E108 interacts with N64, and L1 is in a dynamic released conformation, enabling it to interact with the σ^54^ substrate (16). Subsequently, it was discovered that the ‘Glutamate Switch’ pair is conserved across AAA+ proteins (3,28). In addition to regulate substrate binding through nucleotide binding and hydrolysis, the ‘Glutamate Switch’ could also regulate ATP hydrolysis upon binding to its remodeling target (3).

To gain insights into the roles of nucleotide dependent actions of AAA+ bEBPs and their regulations, we sought variants of a well studied bEBP, the PspF protein that are capable of remodeling its target RP_c_ but have altered interactions across the ‘Glutamate Switch’ pair (E108-N64). We also studied *in trans* regulation of PspF ‘Glutamate Switch’ variants by their negative regulator PspA. Here we report the functional characterizations of ‘Glutamate Switch’ variants and in particular E108Q, leading to the proposal that bEBPs act as templates to drive towards and capture rare and otherwise unstable conformations of RNAP-σ^54^ that are favorable for RP_o_ formation. We show that the distinct states of bEBPs correlate with functional steps in the transition from RP_c_ to RP_o_ (Figure 1B). Specifically, substrate engagements lead to the removal of inhibition on RP_o_ formation imposed by σ^54^ while hydrolysis *per se* is required to remodel the duplex DNA that leads to transcription bubble formation. Furthermore, by analyzing single chain forms of PspF with a combination of wild-type (WT) and variant amino acid sequences in adjacent AAA+ subunits, we conclude that the mechano-chemical coupling in bEBPs requires distinct activities of several of the AAA+ subunits.

## MATERIALS AND METHODS

### *In vitro* spRNA synthesis assay

In a 10 μl reaction with 1x STA buffer (2.5 mM Tris-acetate pH 8.0, 8 mM Mg-acetate, 10 mM KCl, 1 mM DTT, 3.5% (w/v) PEG 8000), 100 nM holoenzyme (reconstituted at a ratio of 1:4 core: σ^54^), 0–6 mM ATP (or dATP) and 20 nM DNA template, was incubated for 10 min at 37°C. This was followed by the subsequent addition of the PspF_1–275_ variants (5 μM), which were incubated for a further 10 min at 37°C. Synthesis of short primed RNA (spRNA) was initiated by adding a mix containing 100 μg/ml heparin, 0.5 mM UpG and 4 μCi [α-^32^P] GTP and incubated for 20 min at 37°C resulting in the formation of the spRNA product 5′-UpGGG. The reaction was quenched by addition of loading buffer and analyzed on a 20% poly-acrylamide denaturing gel, and visualized and quantified using a Fuji FLA-5000 PhosphorImager. All experiments were minimally performed in triplicate.

### Full-length transcription

In a total reaction volume of 10 μl, transcription assays were performed in STA buffer with 10 nM supercoiled template pMKC28 (which contains a T7 early transcriptional terminator sequence ∼470 nucleotides downstream from the multiple cloning site) and 100 nM holoenzyme (reconstituted in a 1:4 ratio of core RNAP: σ^54^). For open complex formation 5 mM PspF_1–275_ and 4 mM ATP or dATP (to limit the role of PspF to RP_c_ formation, since dATP is not an efficient substrate for RNAP) were used. The elongation mixture contained 0.1 mg/ml heparin, 100 nM ATP, CTP and GTP and 50 nM UTP (0.074 μCi/μl of [a-^32^P] UTP (3000 Ci/mmol)). Reactions were incubated at 37°C and stopped with 4 μl of formamide dye mixture (0.3 mg/ml xylene cyanol, 0.3 mg/ml bromophenol blue, 20 mM EDTA in deionised formamide). 7 μl of the samples were run on a 4% denaturing sequencing gel for 2 h at 50 W. The gels were then dried and quantified and analyzed by PhosphorImager analysis to measure transcriptional activity compared to WT and the varying nucleotide conditions for hydrolysis by PspF variants. All experiments were performed minimally in triplicate.

### Gel Filtration

Forty microliters samples were prepared in running buffer (20 mM Tris-HCl pH 8.0, 50 mM NaCl, 15 mM MgCl_2_, with or without: 0.4 mM ATP; 0.02 mM ATP) and centrifuged at 15 000 rpm for 3 min (4°C) to remove any particulates. The samples were pipetted into 200 μl crimp autosampler vials. Each sample was placed in the refrigerated autosampler of the Thermo Scientific Surveyor HPLC system. The Biosep Sec-S3000 gel filtration column (Phenomenex) was attached to the system in a column oven (Phenomenex) at a temperature of 8°C. The detector was set to detect broad spectrum and UV at 280 nm. The flow rate was set at 0.5 ml/min with a pressure limit of 1500 psi and the injections were set at 15 μl. Security Guard (Phenomenex) was used as the guard column to protect the Biosep column from particulate damage.

### ATPase assay

#### NADH-coupled ATPase assay

The steady-state ATPase activity of PspF_1–275_ was measured at 37°C using an NADH-coupled ATP regeneration system (Norby, 1988). Reactions were conducted in 100 μl volumes: 25 mM Tris–HCl (pH 8.0), 100 mM KCl, 10 mM MgCl_2_, 1 mM DTT, 1 mM NADH, 10 mM phosphoenolpyruvate, 10 U/ml pyruvate kinase, 20 U/ml lactate dehydrogenase, 0.02 mM ATP, ±10 μM σ^54^ and 1–5 μM PspF_1–275_. The rate of NADH absorbance decrease at 340 nm is proportional to the rate of steady-state ATP hydrolysis. Assays were performed minimally in triplicate.

#### TLC plate ATPase assay

Typically in a 10 μl volume, 4 μM PspF_1–275_ was preincubated with the ATPase buffer (20mM Tris-HCl pH 8.0, 50mM NaCl, 15mM MgCl_2_, 0.1mM EDTA, 10 mM DTT) at 37°C for 5 min. ATP hydrolysis was initiated by addition of 1mM unlabeled ATP and 0.6 mCi/ml [α-^32^P] ATP (3000 Ci/mmol) and incubated for various time spans at 37°C. Reactions were quenched by addition of 5 volumes of 2 M formic acid. The [α-^32^P] ADP was separated from the [α-^32^P] ATP by thin layer chromatography (Macherey–Nagel) in 0.4 M K_2_HPO_4_/0.7 M boric acid. Radioactivity was scanned by PhosphoImager and analyzed by Aida software. Assays were performed minimally in triplicate.

### Native gel mobility shift assay

Native gel mobility shift assays were conducted in STA buffer in a total reaction volume of 10 μl containing 200 nM RNAP-σ^54^ (reconstituted using a 1:4 ratio of RNAP:σ^54^) and 20 nM ^32^P-labeled probe, which was incubated for 5 min at 37°C. Reactions were analyzed using a 4.5% native (nondenaturing) gel run at 100 V for 55 min. The gels were dried and protein–DNA complexes were visualized and quantified using an FLA-5000 PhosphorImager. These experiments were minimally performed in triplicate.

### Protein purification, crystallization and nucleotide soaking

PspF_1–275_E108Q was over expressed and purified as described previously (11). Crystals of PspF_1–275_E108Q were grown in a sitting drop vapor diffusion experiment at 18°C with 40 mg/ml protein concentration in a precipitant consisting of 12–16% MPD, 2 M ammonium formate and 100 mM Bis-tris Propane, pH 8.0. For APO datasets, the native crystals were soaked in a buffer containing 30% (w/v) PEG8000, 30% (w/v) glycerol and 100 mM HEPES, pH 8.0 before flash freezing in liquid nitrogen. For Mg-ATP and ADP bound structures, native crystals were soaked in 30% (w/v) PEG8000, 30% (w/v) glycerol, 100 mM HEPES, pH 8.0 with 30 mM MgCl_2_ and 25 mM ATP/ADP for 2 h at room temperature and then flash frozen in the same buffer in liquid nitrogen.

### Crystallographic data collection and processing

Data were collected under cryogenic conditions (100 K) at the Diamond Light Source (DLS, UK) at beam lines I02 for nucleotide soaked crystals and I04 for APO crystals. All the datasets were processed using XDS (29) and the statistics are summarized in Table 1.

**Table 1.** Crystallographic data and refinement statistics

	Apo-PspF^E108Q^	Mg-ATP-PspF^E108Q^	ADP- PspF^E108Q^
Space group	*P*6_5_	*P*6_5_	*P*6_5_
Unit cell (Å)	*a* = *b* = 113.72, *c* = 39.45	*a* = *b* = 113.55, *c* = 39.51	*a* = *b* = 113.38, *c* = 39.33
	*α* = *β* = 90°, *γ* = 120°	*α* = *β* = 90°, *γ* = 120°	*α* = *β* = 90°, *γ* = 120°
A. Data reduction statistics			
*λ* (Å)	0.9794	0.9794	0.9794
Resolution (Å)	28.43–1.63	28.39–1.54	28.35–1.42
	(1.67–1.63)	(1.59–1.54)	(1.47–1.42)
Total/unique reflections	303476/36572	426776/43437	538980/54854
	(26792/3380)	(39289/4261)	(51234/5428)
Redundancy	8.3 (7.9)	9.8 (9.2)	9.8 (9.4)
I/*σ*	21.1 (2.08)	23.9 (3.2)	28.7 (3.3)
Completeness (%)	99.35 (93.34)	99.85 (98.54)	99.96 (99.63)
*R*-meas	0.06 (0.89)	0.052 (0.81)	0.042 (0.62)
CC(1/2)	1 (0.73)	0.999 (0.88)	1 (0.87)
Wilson B-factor	23.35	21.67	18.58
B. Refinement Statistics			
Reflections (work/free)	34746/1826	41255/2182	52102/2752
Number of non hydrogen atoms/ water/ligand	2095/174/21	2203/213/59	2211/241/48
*R*_work_ (%)	0.172 (0.257)	0.147 (0.189)	0.169 (0.235)
*R*_free_ (%)	0.204 (0.283)	0.179 (0.265)	0.189 (0.261)
Ramachandran favoured (%)	98	98	99
Ramachandran outliers (%)	0.42	0	0
RMS (bonds) (Å)	0.008	0.01	0.012
RMS (angles) (°)	1.17	1.14	1.35
Average *B*-factor	31	29	25
(Macromolecule/ligand/water)	(30/51/45)	(28/44/41)	(23/33/40)

### Structure determination

Phases for the datasets were obtained using Phaser (30) resulting from molecular replacement solutions, with the native WT PspF structure as the search model (2BJW) (11). Refinement of the models was carried out using phenix.refine implemented in the software suite PHENIX (31). Cross-validation was performed using 5% of the dataset set aside for R_free_ calculations. Electron density for the nucleotide analogues ATP and ADP was clearly visible after an initial round of refinement using the molecular replacement solution of the native structure and the ATP and ADP were then accordingly placed into density. Clear density was also visible for Mg^2+^ in the Mg-ATP structure and was thus placed. Refinement statistics for all the structures are summarized in Table 1.

## RESULTS

### The effects of the Walker B residue E108 are highly dependent on DNA substrates

In order to investigate the precise functionality of the Walker B residue E108, which was proposed to coordinate the hydrolytic water molecule for in-line nucleophilic attack (2), we carried out transcription assays using WT, E108A, E108D and E108Q variant proteins in the presence of different nucleotides and in the presence of a DNA duplex (mimicking DNA substrate in RP_c_) or with mismatches from −10 to −1, mimicking the transcription bubble, which is found in a fully formed RP_o_ (Figure 1) (as in (23)). The E to A mutation removes the side chain of the residue, E to D maintains the negative charge property but with a shortened side chain while E to Q change maintains the side chain geometry except the OH of one side chain branch is replaced by a NH_2_ group. In addition to different DNA substrates, we also challenged the reactions with different nonhydrolysable nucleotide analogues, in order to probe different functional states during ATP hydrolysis cycle. Remodeling activities of transcriptionally competent complexes were monitored by 5′-UpGGG transcript formation primed with the dinucleotide primer UpG (Figure 1). Notably, with a standard duplex DNA, only E108D behaves similarly to WT protein, and dATP or ATP (we used dATP as well as ATP to maintain a constant pool of hydrolyzable nucleoside triphosphate for PspF NTPase activity during transcription) is required for transcription activation. All other variant proteins tested are defective in transcription activation using dATP or ATP. However, with the pre-opened DNA substrate, all variants showed transcription activation in an (Nucleoside triphosphate) NTP-dependent fashion although E108A has reduced activities. Strikingly, at low ATP concentration, when WT protein is unable to activate transcription, the E108Q variant not only then activates transcription on duplex DNA but this activity is elevated when the DNA is pre-opened.

### E108Q mutant supports transcription at lower ATP concentrations compared to wild-type even though it has reduced ATPase activity and enhanced ATP binding

**Figure 2.**
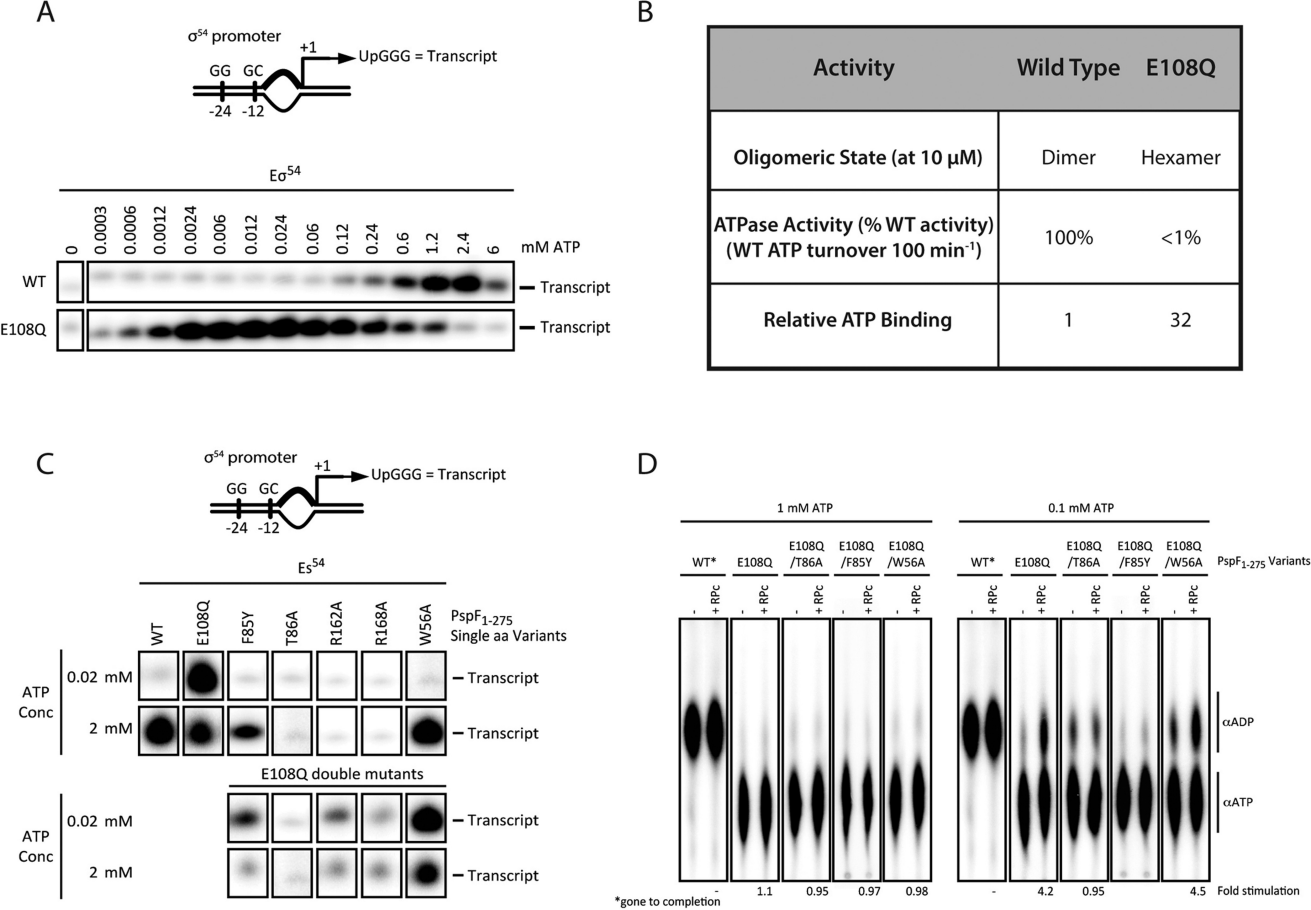
Functional activities of E108Q variant. (**A**) Transcriptional activity dependency of E108Q on ATP concentrations. (**B**) ATP binding and hydrolysis of E108Q compared to wild-type protein. (**C**) The remodeling activity of E108Q compared to other functional mutants. Upper: single mutations, lower: E108Q in combination with other functional mutants as in upper panel. (**D**) ATPase activity of E108Q to probe its dependency on the integrity of the GAFTGA motif by comparing the activity in the absence and presence of RP_c_.

To characterize the ATP dependence of E108Q further, we carried out transcription activation assays for E108Q and WT in a range of ATP concentrations. In agreement with previous reports, the WT has a maximal transcription stimulating activity at around 1–2 mM ATP (Figure 2A). Interestingly, E108Q reaches its maximal activity at 100-fold less ATP concentrations, around 0.02 mM (Figure 2A). Since transcription activation is closely related to ATPase activity, we measured the effects of the Walker B substitution on ATP binding and hydrolysis capability. Not surprisingly, E108Q has severely impaired ATPase activity compared to WT (<1% activity), highlighting the importance of the integrity of Walker B residues. However, it has 30-fold increase in binding affinity to ATP (Figure 2B) (22). These data show that E108Q binds ATP tightly, has poor hydrolysis rate, but can effectively activate transcription using the pre-formed transcription bubble at lower ATP concentration. PspF assemblies have been shown to exist in a mixed nucleotide bound state (32), where both ATP and ADP are bound and proposed to alter the detailed geometry of hexamer favoring its activity in making RP_c_. Further, high ATP concentrations inhibit PspF functionality. The higher affinity for ATP that E108Q possesses compared to the WT PspF suggest that at the E108Q ATPase activity and other functionality could be inhibited at ATP concentrations that are more optimal for WT PspF activities.

Previously, we have shown that the integrity of GAFTGA motif of the L1 is important for PspF to contact RP_c_ (33) while W56 is essential for interacting with its negative regulator PspA, which has been proposed to act through the ‘Glutamate Switch’ (34,35). We show here that the remodeling activity of the E108Q variant was also dependent upon the integrity of the GAFTGA motif of the Loop1 (compare to F85Y, T86A, Figure 2C double mutants) and was independent of W56 (a determinant for binding PspA for the inhibition of PspF) (Figure 2C). Furthermore, despite their overall reduced ATPase activity, the double mutants E108Q-R162A and E108Q-R168A when compared to E108Q display a modest increase in transcriptional activation. This modest increase on loss of the R finger residues may be due to the resulting conformational charge that occurs at the catalytic site. Interestingly, at low ATP concentration, PspA inhibits the activity of E108Q variant and the E108Q-W56A double mutant although the latter inhibition is less severe (Supplementary Figure S1), suggesting that the PspA interaction sites in addition to W56 that exist in PspF are favored in the E108Q variant. Like W56A, N64S has been shown to escape negative regulation by PspA (27). Interestingly, E108Q-N64S double mutant is still under negative control by PspA, further supporting the notion that E108Q mutation promotes inhibitory routes by PspA that are independent of W56.

Since E108 plays important roles in ATP hydrolysis and is part of the ‘Glutamate Switch’ pair (E108-N64) that links substrate binding to ATP binding site, we investigated whether its ATPase activity could be stimulated by binding to its substrate. We added RP_c_ to ATPase reactions. Within a 5-min timescale WT PspF shows no discernible difference (at 1 mM ATP) when supplemented with RP_c_. At 0.1 mM ATP, however, the addition of RP_c_ stimulates ATP turnover by 2-fold (Supplementary Figure S2). It is unclear if this effect is due to a higher affinity e.g. ATP or more efficient phosphate release. Although WT shows a 2-fold stimulation by binding to its substrate, the E108Q variant shows a 4-fold stimulation upon binding to RP_c_ at the lower ATP concentration (0.1 mM) and this stimulation depends on substrate interaction via the GAFTGA motif as mutating T86A, which is shown to impair its interactions with the RP_c_ target remodeling substrate abolishes the stimulatory effects (Figure 2D). The double variants E108Q-T86A and E108Q-W56A both display an increase in ATPase activity compared to E108Q (Figure 2D). These observations are consistent with previous studies that implicate the connection of the L1 loop and W56 to the E108-N64 pair as a means of hydrolysis control (16,27).

### E108Q forms constitutive hexamers and alternative competitor resistant complexes

**Figure 3.**
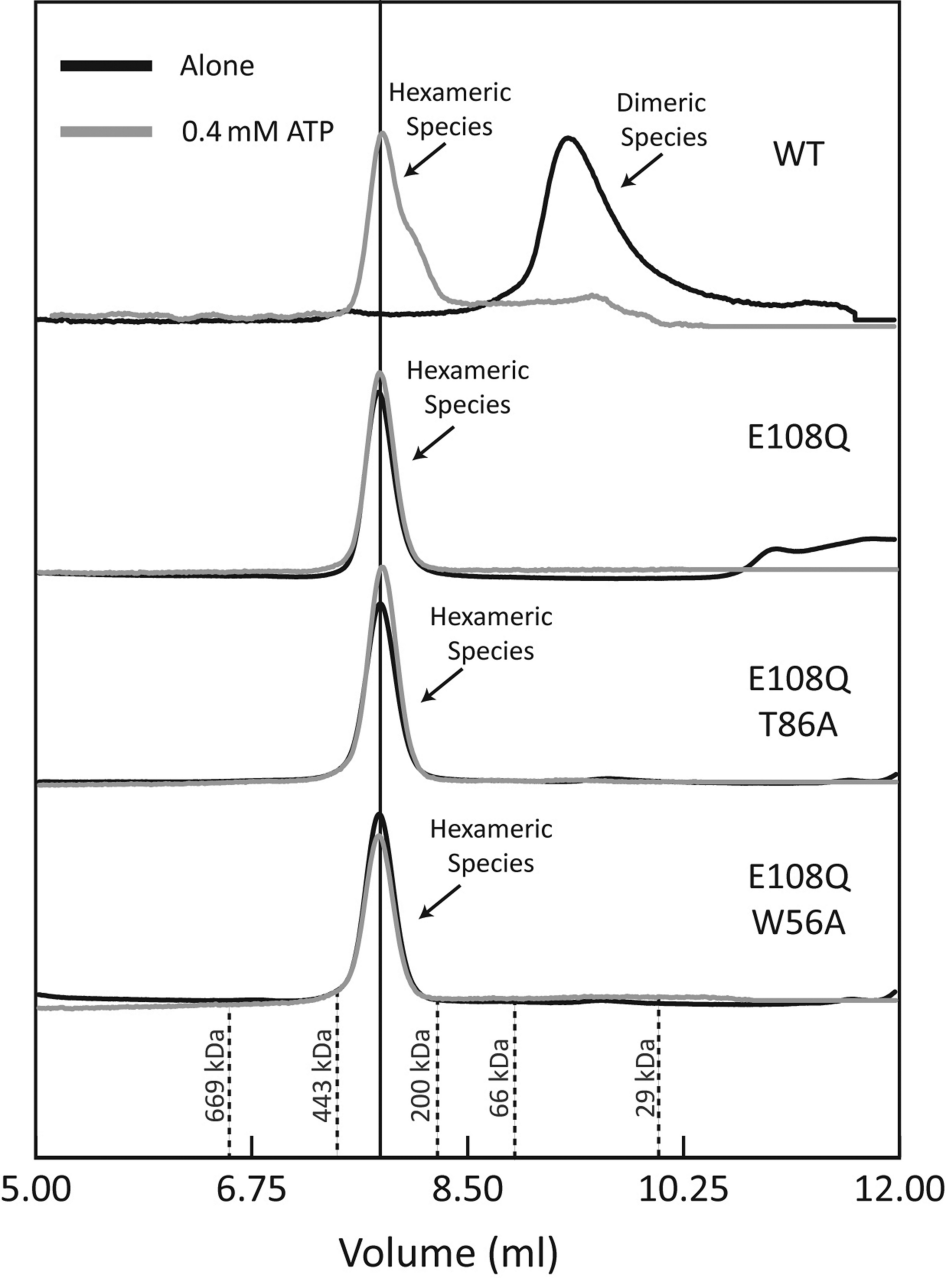
E108Q forms constitutive hexamers. Gel filtration profile of E108Q mutant protein compared to wild-type protein. WT PspF AAA domain predominantly exists as dimers in the absence of ATP, which is required for hexamer formation. PspF AAA domain E108Q variant exists as hexamers irrespective of nucleotide and the hexamers are not dependent on the integrity of RNAP-σ54 binding site (T86A in Loop1) nor the PspA inhibitor binding site (W56A).

Hexamerization has been linked to nucleotide binding and is one pre-requisite for ATP hydrolysis by PspF (5). To investigate if the reduced ATPase activity of E108Q is due to impaired hexamerisation, we assayed the oligomeric state of PspF variants using gel filtration (Figure 3). For WT proteins, in the absence of nucleotide, PspF exists primarily as dimers at low μM concentrations and upon ATP binding, PspF shifts to a predominately hexameric peak. E108Q, on the other hand, is hexameric irrespective of the nucleotide being present, suggesting that the reduced ATPase activity is due to a reduced hydrolysis reaction *per se*, and not due to gross defects in hexamerisation nor loss of nucleotide binding. The nucleotide analogue ADP-AlF has been traditionally used to capture the RNAP-σ^54^–DNA intermediate complex (RP_i_) (Figure 1B). E108Q, however, forms an RP_i_ in the presence of ATP but not in the presence of ADP or in the APO state (Supplementary Figure S3).

**Figure 4.**
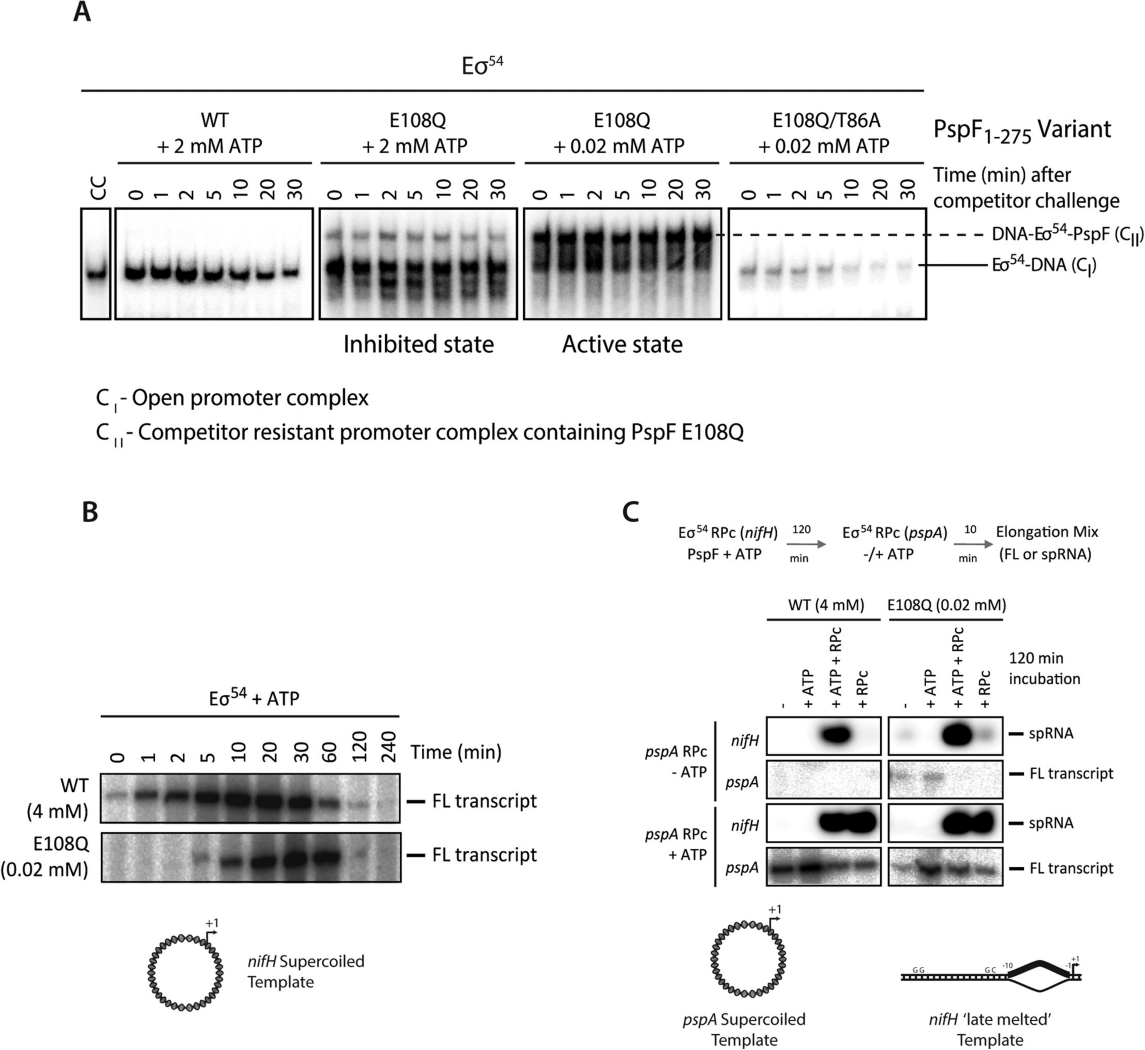
E108Q promotes the formation of a transcriptional intermediate complex en route to open complex formation and E108Q can support persistent transcription. (**A**) Heparin resistance of complexes between PspF and the RP_c_ as assayed by native gels. (**B**) E108Q can support full length transcript formation. (**C**) Transcripts from two different promoters (nifH and pspA). To probe the remaining transcription capacity of PspF wild-type (WT) and E108Q variant, the RP_c_ with *pspA* template was added after the activation and transcription reactions had taken place on the *nifH* probe. After 120 min incubation, E108Q protein remains competent for full length transcript formation without further added ATP while WT protein requires additional ATP.

E108Q clearly has altered properties in ATP binding and hydrolysis, hence altered kinetics of the ATP hydrolysis cycle. It is therefore potentially capable of forming altered intermediate transcriptional complexes with its RP_c_. To probe for these, we challenged the transcription reactions with the competitor heparin. Normally RP_c_ is heparin sensitive while RP_o_ is resistant. Interestingly, two distinct complexes formed for E108Q protein depending on the level of ATP, and these complexes are not observed for WT protein (Figure 4A). The magnitude of the shifts and the ability of ATP to stabilize complexes between σ^54^ and PspF E108 variants suggest that these gel mobility results were due to the presence of E108Q within the new complexes. At the non-inhibitory low ATP levels, a slower migrating complex containing E108Q is the predominant species and its formation is dependent on an intact GAFTGA motif (which contacts σ^54^) implying specificity and exposure and stabilization of the L1 loop (Figure 4A E108Q/T86A double mutant) and that the new co-complex is stable under heparin challenge (100 μg/ml). Notably these complexes are distinct from RP_c_ (by their stability to heparin) and the RP_o_ (in terms of gel mobility) and so are most likely transcription intermediates containing the E108Q variant and lying between RP_c_ and RP_o_ on the pathway to transcription initiation. At higher ATP concentrations, a complex corresponding to RP_o_ dominates although other fast migrating bands are also seen which are consistent with PspF oligomers. This suggests that at inhibitory ATP concentrations, significant portion of PspF E108Q mutant protein dissociates from RNAP-σ^54^–DNA complex, consistent with the inhibitory effects at high ATP concentrations.

We considered whether the accumulation of transcription intermediates with E108Q might hinder steps after RP_o_ formation such as promoter escape. However E108Q at low ATP concentrations supported full length transcript formation, and did not prevent promoter escape (Figure 4B). Full length transcript assays showed that although E108Q exhibited a delayed activation compared to WT protein, and that E108Q displayed a persistent transcription stimulating activity as shown in the time course experiments (Figure 4B). Furthermore, our data show that the E108Q mutant protein could support full length transcript formation at least at the pspA promoter site, without addition of ATP after 120 min incubation with 0.02 mM initial ATP whereas WT protein did not with 4 mM initial ATP (Figure 4C, compare the boxed areas). Together these data support the idea that E108Q is a slow but stable productive ATPase compared to the faster WT PspF.

Previously, using pre-formed transcription bubble substrate, the ADP.AlF nucleotide analogue was shown to support a partial remodeling of the RNAP–σ^54^ holoenzyme but not the extensive *de novo* DNA melting as seen in RP_o_ nor full length transcription, suggesting that RNAP-σ^54^ engagement at the ATP hydrolysis transition state can partially overcome transcription suppression imposed by RNAP-σ^54^ (23). Our results here with E108Q show that RNAP-σ^54^ engagement can also remodel the RNAP-σ^54^ holoenzyme to such an extent that it can overcome suppression to support full transcription persistently. Unlike the ADP.AlF bound pre-initiation complex, which can only partially activate transcription, E108Q variant can fully activate transcription (comparing the activities between WT and E108Q) when DNA is pre-melted, suggesting that E108Q variant represents a functional state further along the activation pathway compared to those represented by ADP.AlF bound complex. The slow ATPase activity of E108Q allows the detection of such an intermediate state that might be short-lived with WT PspF. Our data here thus support a model where the set of distinct kinetic steps in transcription activation intimately involve the functional states of the activator protein, driving the conversion of RP_c_ to RP_o_.

### E108Q variants support functional asymmetry required for PspF activities

**Figure 5.**
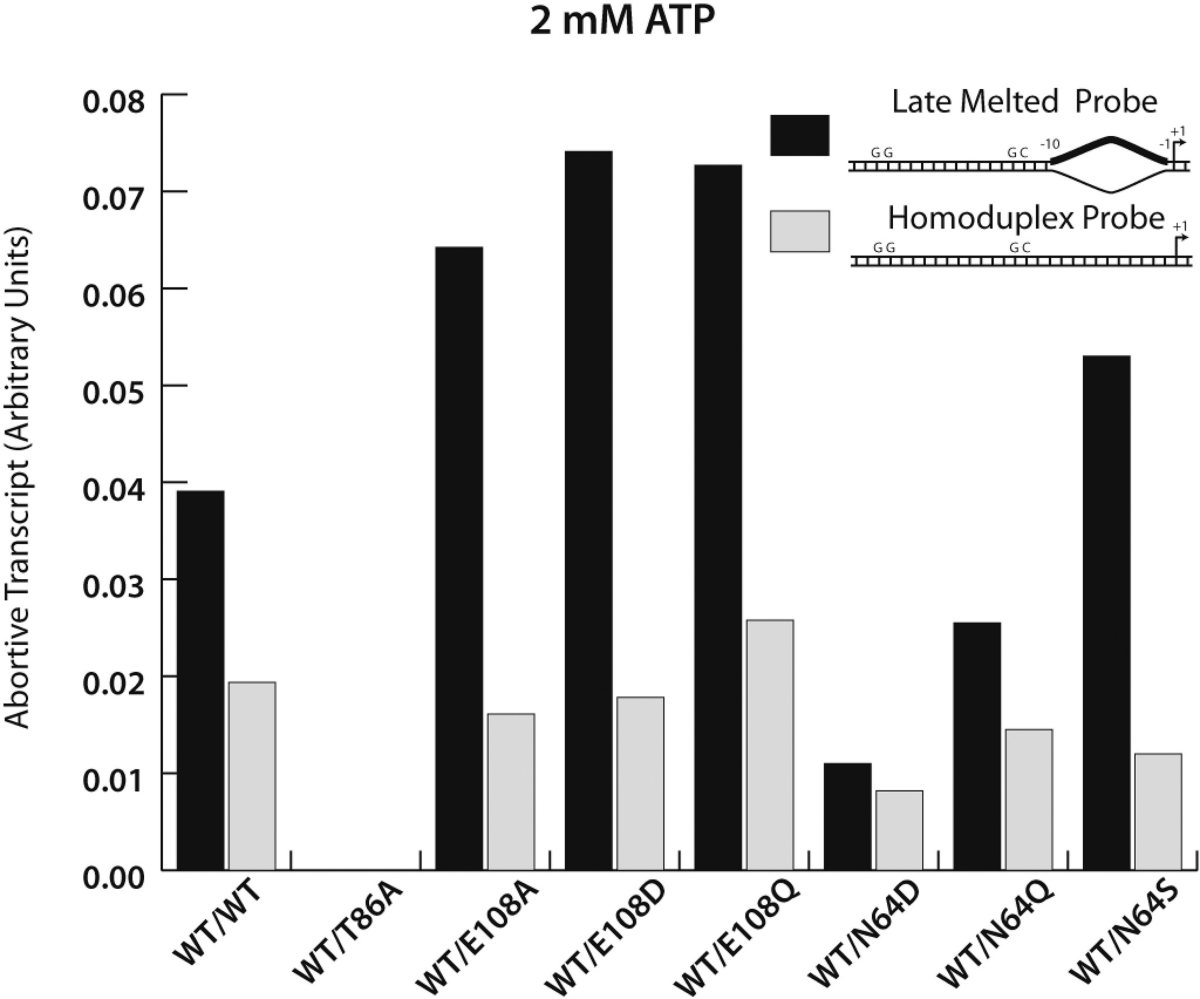
Activities of single chain forms of PspF variants. Alternating wild-type with glutamate switch mutants (E108 or N64 variants) within the hexamer result in forms that favor protein remodeling over DNA remodeling.

To explore further subunit specializations within hexameric bEBP assemblies, we constructed single chain forms of the PspF AAA+ domain with alternating WT and E108Q, E108D or E108A substitutions (36). A linked dimer form of PspF was constructed by adding a linker sequence between the C and N terminal residues of two PspF AAA domains and expressing the linked coding sequences as a single polypeptide chain (36). By assaying these constructs against fully duplexed or opened up DNA templates bound by RNAP-σ^54^ we show that E108Q, E108D and E108A, even when coupled to WT subunits, greatly favors transcription activation with pre-formed transcription-bubble over the duplex DNA substrates (Figure 5, Supplementary Figure S4). E108Q, E108D and E108A can all support transcription activation with pre-formed transcription bubble (Figure 1). E108Q, E108D and E108A are shown to have reduced ATPase activity but can still use ATP to engage with RP_c_. T86A in Loop1, on the other hand, does not alter ATPase activity but fails to engage with RP_c_ (11). As expected, T86A cannot efficiently support transcription even when a subset of the subunits are WT (Figure 5) because two adjacent L1 loops need to be fully functional for binding to RP_c_. The ‘Glutamate Switch’ variant N64S, which is shown to be able to engage with the RNAP-σ^54^, can also support transcription and greatly favors pre-formed transcription bubble over the duplex DNA. These outcomes imply that transcription activation can be supported by a fraction of the ATPase subunits, some of which may have defective ATPase activity but can associate with the target RP_c_ such as in WT/E108Q variant. However, subunits that are relatively fast for ATPase activity but do not stably associate with RP_c_, as shown in WT/T86A, are insufficient for transcription activation. These results highlight that multiple subunits with intact L1 loops are required to engage with the RNAP-σ^54^ (as shown in WT/T86A) for efficient remodeling although stable association with a subset of subunits (WT/E108Q) showing slow ATPase are sufficient. Interestingly, despite the defective ATPase activity of PspF E108Q, the ATPase activity of WT/E108Q linked dimer is WT-like at the higher ATP concentration (1 mM) but E108Q-like at lower ATP. This is in agreement with a model in which mixed nucleotide states exist but in a highly coordinated fashion within a hexameric PspF. It is possible that E108Q subunits adopt certain conformations that are coupled to adjacent subunits, which then adapt conformations competent for using ATP in remodeling the RP_c_. This model can also explain the elevated ATPase activity of the linked dimer at the lower ATP concentration (0.1 mM) compared to WT/WT (Supplementary Figure S5) as the increased ATP affinity of E108Q would promote efficient conformational coupling to its adjacent WT subunits.

### Crystal structures of PspF_1–275_ E108Q mutant in APO, ATP and ADP states

In order to understand the biochemical properties of E108Q and to provide a structural basis for the ‘Glutamate Switch’ pair, we determined the crystal structures of E108Q mutant of PspF AAA+ domain (PspF_1–275_E108Q) in the absence of nucleotide (APO), and in the presence of Mg-ATP or Mg-ADP. The crystals diffracted to 1.6–1.8 Å and have the same space group and unit cells as those of WT proteins and are thus subsequently rebuilt and refined using WT protein structures as templates. The crystallographic data and refinement statistics are summarized in Table 1.

**Figure 6.**
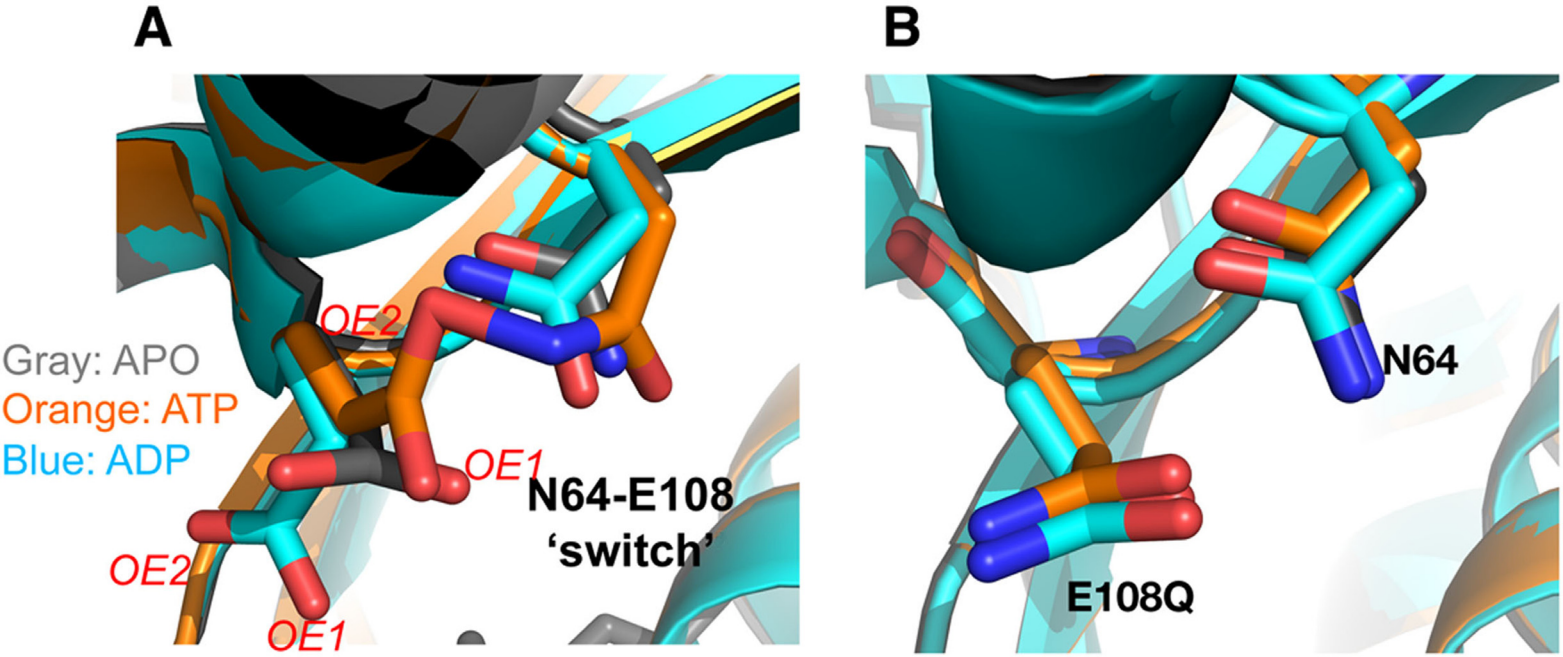
Structural changes of the ‘Glutamate Switch’ pair. (**A**) In wild-type (WT) protein, the E108 adapts different conformations depending on the nucleotide states. (**B**) In E108Q variant protein, irrespective of nucleotide bounds states, E108Q adapts a conformation similar to the APO/ATP bound conformation in the WT protein, where the Glutamate Switch pair is engaged. Gray—apo, orange—ATP, cyan—ADP. OE1 and OE2 refer to the two different glutamate oxygen atoms.

We first compared the conformations surrounding the ‘Glutamate Switch’. In WT protein, when ADP is bound, E108 does not interact with N64 albeit in the ATP bound or APO state, E108 interacts with N64 (Figure 6A, (16)).

In the mutant structures, on the contrary, E108Q in the ADP bound state as well as in the APO state also interacts with N64 (Figure 6B). This conformation, which enables the ‘Glutamate Switch’ pair to interact, is linked to the ability of PspF being able to engage with its target, the RP_c_. The crystal structures of the E108Q therefore suggest that PspF E108Q variant could engage with RP_c_, irrespective of its nucleotide bound states. Such a conformation could contribute to the ability of E108Q to bind RP_c_ and move RP_c_ along the pathway of transcription activation despite its slow ATPase activity.

To further investigate the structural basis for these distinct conformations, we analyzed the detailed interaction networks surrounding these key residues in the different nucleotide bound states, both in the WT and in the mutant protein structures.

**Figure 7.**
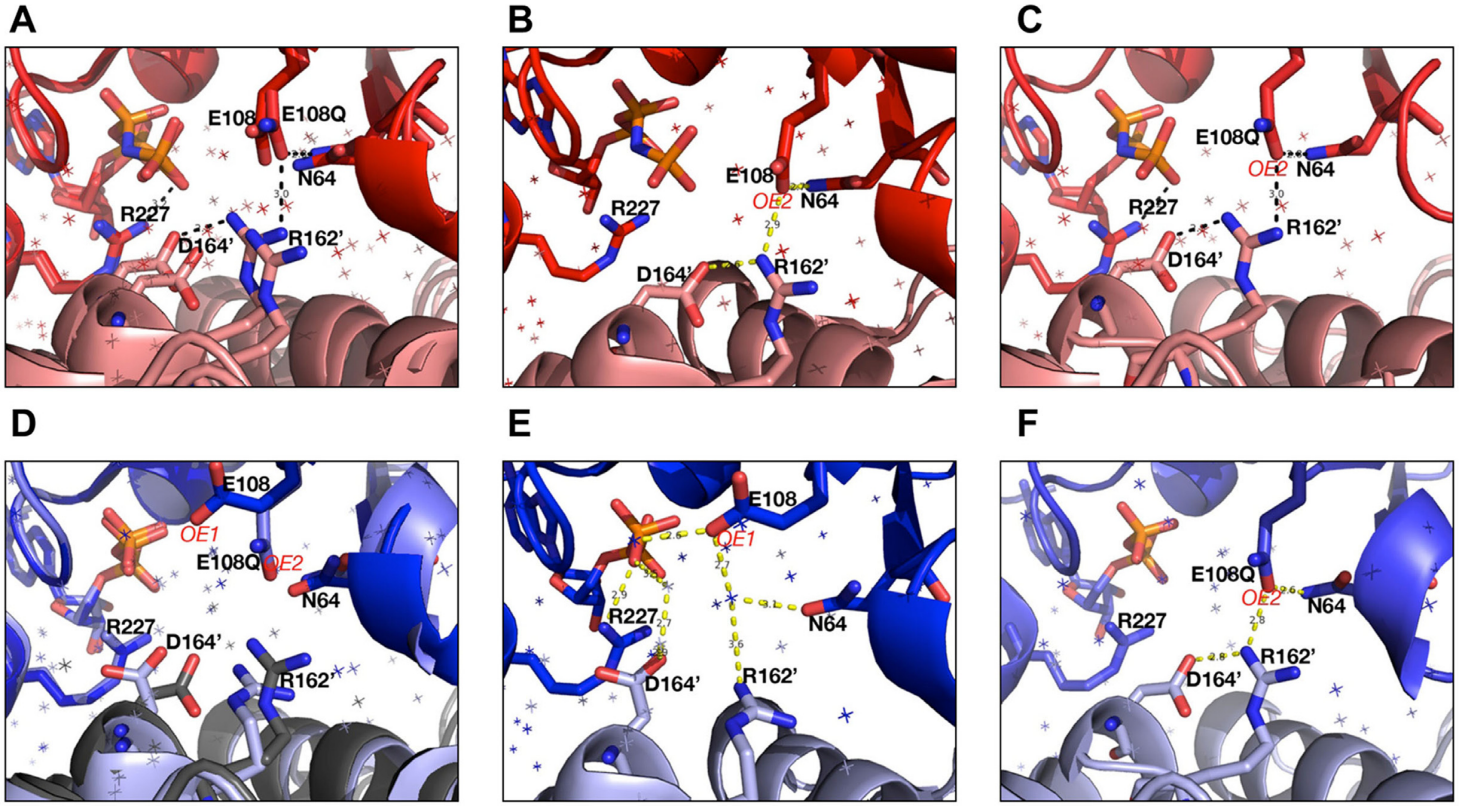
Structural comparisons of the nucleotide binding pockets between wild-type (WT) and E108Q mutant proteins. Red—ATP bound states, blue—ADP bound states. (**A**) Superposition of WT and E108Q ATP bound states. (**B**) WT ATP states showing key interactions involving E108 OE2. (**C**) E108Q ATP state showing key interactions. (**D**) Superposition of WT and E108Q ADP states. (**E**) WT ADP state showing key interactions involving E108 OE1 which is mutated in E108Q. (**F**) E108Q ADP state showing interactions involving OE2 instead.

The WT and E108Q mutant structures are almost identical surrounding the ‘Glutamate Switch’ pair (Figure 7 and also (16). The ‘Glutamate Switch’ pair is located at the interface of two neighboring protomers (Figure 5 and 7A). Similar residues are involved in the interaction networks and the interactions are mainly electrostatic involving E108, N64 as well as residues from adjacent protomer including the R finger residue R162′ and D164′ (′denotes adjacent protomer). Specifically, the oxygen atom (OE2 in Figure 7B) of E108 interacts with NH_2_ groups of N64 and R162′ from adjacent protomer. R162′ also interacts with D164′ (Figure 7A–C). These interactions are similarly maintained in the mutant structure since the mutation does not affect the oxygen (OE2) of residue 108.

A direct superposition of the mutant and WT ADP bound structures reveal two major differences surrounding the ‘Glutamate Switch’ pair. In addition to the distinct conformation of E108 and E108Q, D164′ from adjacent protomer, another highly conserved residue in bEBPs, also adapts distinct conformations (Figure 7D). In the WT structure, there are a number of water molecules in the nucleotide binding pocket, mediating a network of hydrogen bonding interactions involving E108, N64 and the sensor II R227 as well as residues from the adjacent protomer including R162′ and D164′ (see Figure 7E). In particular, E108 and D164′ only form hydrogen bond interactions with water molecules. However, in the mutant protein, the OH group in E108 (OE1 in Figure 7E) that is involved in hydrogen bonding is replaced by an NH_2_ group in E108Q, no longer able to maintain a stable hydrogen bond network with these water molecules. Instead, E108Q returns to a conformation optimal for interacting with N64 through OE2 of E108Q (Figure 7F). The loss of water mediated hydrogen bonding network also destabilizes the D164′ side chain conformation. D164′ side chain rotates to interact with R162′. Consequently, in this conformation, R162′ makes charge–charge interactions with both E108Q and D164′ (Figure 7F), resulting in distinct side chain conformations in ADP bound states between WT and E108Q mutant proteins.

## DISCUSSION

### A structural basis for the precise role of E108 and the distinct properties of E108 variants

Our data on the formation of a distinct transcriptional intermediate complex with E108Q mutant show that a range of RNAP-σ^54^ engagements can be obtained when ATP hydrolysis is slowed down. The significantly impaired ATPase activity of E108Q mutant and its ability to activate transcription when a transcription bubble is pre-formed imply that efficient RNAP-σ^54^ engagement is sufficient for removing transcription inhibition but not sufficient for extensive *de* *novo* DNA opening. ATP hydrolysis and the associated conformational changes in the AAA domains of at least two adjacent subunits with a hexameric assembly are required for this DNA remodeling. Strikingly, competitor resistant transcriptional intermediates are evident with E108Q (Figure 4A), and these intermediates can be correlated with persistent transcription of E108Q at lower ATP concentration (Figure 4B) or even without further ATP addition after a prolonged incubation with RP_c_ (Figure 4C).

It appears that the E108Q variant is competent at engaging RP_c_ in a nucleotide bound state prior to ADP + Pi release. This engagement property of E108Q can be explained by considering the altered interaction networks in its nucleotide bound forms compared to the WT. These changes are mainly due to the hydrogen bonding networks mediated via water molecules and involve the OH group of one of the E108 side chain branches. Mutating this OH to NH_2_ would thus destabilize the water-mediated hydrogen bonding network, irrespective of the nucleotide bound state. This explains its ability to engage stably with RNAP-σ^54^, hence its favored ability in activating transcription from a pre-opened transcription bubble and persistent transcription under competitor challenge conditions.

The single chain form of PspF shows that when only a subset of E108Q subunits is present in the hexamer, its ability to remodel DNA is similar to that of WT (Figure 5), implying that DNA opening does not require an equivalent active ATPase activity of adjacent subunits. In low ATP concentration conditions, WT/E108Q does not activate transcription to the same level as E108Q (Supplementary Figure S4), suggesting that even though active ATPase activity is not required for adjacent subunits, ATP binding to adjacent subunits is required for transcription activation. WT has 30-fold lower affinity for ATP, correlating with its optimal ATP concentration being 100-fold higher than that of E108Q variant. These results support a model in which PspF does not function as a processive motor. Rather a bimodal switch function of PspF requiring the cooperation of several neighboring subunits, at least for ATP binding, seems to operate. This correlates with the requirement of stable RNAP-σ^54^ engagement by several adjacent subunits to remodel RP_c_ and activate transcription. Furthermore, defects in ‘Glutamate Switch’ mutants (such as those E108 or N64 mutants, Figure 5) results in a form of PspF that favors protein remodeling over DNA remodeling, supporting the idea that stable RNAP-σ^54^ engagement is sufficient to remove the suppression imposed by RNAP-σ^54^ on protein organization for RP_o_ formation while full ATP hydrolysis and associated conformational changes are required for the DNA melting and transcription bubble formation seen in an RP_o_.

### Implications for isomerisation

Our results reveal and explain the differences in the ATP binding and hydrolysis properties between the WT and the E108Q variant proteins. The preferred conformation of E108Q in the ‘inactivated’ form could also explain the significantly enhanced affinity for ATP (32-fold increased compared to WT) and the ability to readily form hexamers. However, due to its reduced stability in the ‘for hydrolysis activated’ form, the ability to hydrolyse ATP is significantly impaired (<1% compared to WT). The constitutive hexamer formation in E108Q and reduced ATPase activity could also explain the observed stimulatory effects on ATPase activity in E108Q upon RNAP-σ^54^ binding, which re-orients the E108Q to be in the ‘for hydrolysis activated’ form. In WT proteins, the ATPase activity is affected by hexameration which in turns is affected by ATP and RNAP-σ^54^ binding. Furthermore, the high base level of the ATPase activity in the WT protein suggests a preferred orientation of E108 in the ‘for hydrolysis activated’ form, hence the limited stimulatory effect upon RNAP-σ^54^ binding.

Interestingly, in WT, the optimal ATP concentration for ATP hydrolysis as well as transcription activation is at 1–2 mM while the E108Q variant requires significantly less ATP (0.02 mM) for its optimal function even though the ATP hydrolysis rate is much slower. This reduced ATPase is partially restored due to the stimulatory effects upon binding to the RP_c_. These results also re-enforce that RP_c_ engagement, not remodeling due to hydrolysis *per se*, is sufficient in removing the inhibition to protein conformational change in RP_c_, which forms a major kinetic barrier in isomerisation to make the RP_o_. Due to the 30-fold increased affinity for ATP, significantly lower ATP concentrations are thus required for stable RP_c_ engagement. Interestingly, mutations in the ‘Glutamate Switch’ N64 have similar phenotypes to those of E108 in favoring the ‘pre-opened transcription bubble’ as a transcription substrate (Figure 5), suggesting that the ability for the ‘Glutamate Switch’ to form and dissociate are key to DNA opening. Target engagements can be achieved when ‘Glutamate Switch’ pair is formed as demonstrated with E108Q mutant protein, which readily forms hexamers and binds to ATP, and is able to engage with the RP_c_ complex for a prolonged period of time ahead of ATP turnover. The reduced hydrolysis rate would reduce the speed for the final conformation changes required for transcription bubble formation but favor protein remodeling (23). The particular properties of the E108Q variant provide evidence for a set of distinct kinetic steps along the activation process. The altered dynamics between the different steps due to the E108Q mutation seems to result in an overall more efficient use of ATP in this process, but as noted above the penalties are defects in DNA opening and a strong ATP inhibition and a slow conversion of RP_c_ to RP_o_. In physiologically conditions, the 2–3 mM concentrations of ATP in *Escherichia coli* suggests that E108Q, though a functional ATPase variant, is unlikely to be viable to support transcription.

## PDB ACCESSION CODES

4QNM: PspF1-275E108Q mutant; 4QNR: PspF1-275E108Q mutant bound to ATP; 4QOS: PspF1-275E108Q mutant bound to ADP.
